# EHMTI-0110. Epidemiology of primary headaches in the population of Baku

**DOI:** 10.1186/1129-2377-15-S1-B14

**Published:** 2014-09-18

**Authors:** R Aliyev, R Shiraliyeva, R Hasanov, A Mammadbayli

**Affiliations:** 1 Neurology Department, Azerbaijan State Advanced Training Institute for Doctors named after A.Aliyev, Baku, Azerbaijan; 2 Department of Neurology and Medical Genetics, Azerbaijan Medical University, Baku, Azerbaijan

## Introduction

Headache is one of the most common reasons people see physicians. It has been estimated that 47% of the adult population have headache at least once within last year in general.

That is why the aim of the study was to investigate the prevalence of primary headaches in the population of Baku the capital of Azerbaijan.

## Methods

Baku consists of eleven districts. During 2010-2012 years every 30th of 45,000 Nasimi district habitants were invited to the clinic for an interview and screening of headaches. Only one district habitants were involved to the study. Currently, the total population of district is 202,073 people. The study included 1,300 people. With the help of a standardized questionnaire, have studied the presence of headache, their specificity, the type, risk factors, neurological and somatic status of the respondents, their social and economic status. Diagnosis of headache conducted according to the criteria of the International Classification of Headache (second edition).

## Results

In 40.5% of the respondents in the last 6 months there has been a headache.13.7% of them were men, 26,3% were women. At 18.2% had migraine, 42.2% have tension type headaches. Distribution headaches by sex showed predominance of primary headaches among women (2:1). Primary headaches are other character (cluster, paroxysmal hemicrania, etc.) revealed only a few people that had no practical meaning. The remaining 183 people (34.7%) were diagnosed secondary headaches.

In conclusion our findings may be helpful in organizing the treatment and prevention measures of headaches in Baku.

No conflict of interest.

